# The Cellular Mechanisms that Ensure an Efficient Secretion in *Streptomyces*

**DOI:** 10.3390/antibiotics7020033

**Published:** 2018-04-14

**Authors:** Sonia Gullón, Rafael P. Mellado

**Affiliations:** Departamento de Biotecnología Microbiana, Centro Nacional de Biotecnología (CNB-CSIC), c/Darwin 3, 28049 Madrid, Spain; sgullon@cnb.csic.es

**Keywords:** *Streptomyces lividans*, secretion pathways, secretory proteins, signal peptides

## Abstract

Gram-positive soil bacteria included in the genus *Streptomyces* produce a large variety of secondary metabolites in addition to extracellular hydrolytic enzymes. From the industrial and commercial viewpoints, the *S. lividans* strain has generated greater interest as a host bacterium for the overproduction of homologous and heterologous hydrolytic enzymes as an industrial application, which has considerably increased scientific interest in the characterization of secretion routes in this bacterium. This review will focus on the secretion machinery in *S. lividans*.

## 1. Introduction

In their natural soil environment, the streptomycetes bacteria are characterized by the formation of an aerial mycelium during their life cycle before the sporulation. Along their life cycle, the streptomycetes produce and secrete a variety of hydrolytic enzymes [1,2] and enzyme inhibitors as well as signaling molecules and antibiotics in order to ensure the continuation of their existence in competitive habitats.

Their capacity to produce extracellular homologous and heterologous proteins of industrial application has remarkably increased the interest and targeted research for increasing the knowledge about the functioning of the streptomycetes secretion machinery. Therefore, this information could be used to engineer efficient streptomycetes strains for the overproduction of commercially valuable proteins needed for biotechnological application.

*Streptomyces lividans* possess a relaxed restriction-modification system that facilitates its transformation with exogenous DNA without degrading it. Moreover, it has been a satisfactory potential host for extracellular protein overproduction because of its ability to secrete a variety of hydrolytic enzymes. Additionally, its genome sequence is known [3,4]. The use of *S. lividans* as a host to engineer the production of secretory proteins is well documented [5,6,7].

There are two pathways commonly used to secrete extracellular proteins in *S. lividans.* The Sec route is the major system used to release extracellular proteins through the cellular membrane [8]. A secondary system, also found in *S. lividans*, is the Tat route. The Tat-secreted proteins have the property of appearing fully folded in the culture supernatant [9]. Different types of proteins with the predicted Sec or Tat signal peptides together with the antibiotic undecylprodigiosin and other proteins lacking signal peptides have detected in vesicles present inside droplets found in *Streptomyces* grown on top agar plates. This produces harmful effects in fungi and is a new way to deliver proteins with potentially different applications, which deserves to be explored further [10]. The function of the secretion system known as Esx or Type VII described for the secretion of small size proteins in Gram-positive bacteria [11] is not found in streptomycetes [12].

The characterization of the *S. lividans* cellular response when challenged to overproduce vast amounts of particular secretory model proteins has also been researched in the recent past. This research has provided in-depth information and increased our knowledge of the bacterial factors that may affect the efficiency of secretory protein production by causing different types of stress in the bacterial cell.

## 2. The Sec Pathway

A characteristic signal peptide present in the pre-secretory amino end is the signal recognized by intracellular chaperones to target these proteins to the membrane. A typical signal peptide consists of three regions including a short stretch of positively charged amino acids close to the N terminus (n-region), a much longer stretch of hydrophobic amino acids (h-region), and the region at the end of the signal peptide, which contains the stretch of amino acids that the signal peptidase will cleave (c-region). *Escherichia coli* SecB chaperon prevents pre-secretory protein folding by targeting it to the translocase complex in the membrane by cooperating with SecA [13]. This post-translational secretory protein transport mechanism is widely used for bacterial protein secretion via the Sec pathway. Although genes homologous to SecB are found in Gram-positive bacteria, they are not present in the *Bacillus subtilis* genome [14] or in the genomes of *S. coelicolor* [15] or the genomes of *S. lividans* 66 [3] strains. This fact has triggered more research to find out alternative means that these Gram-positive bacteria use to transport secretory proteins to the membrane.

The signal recognition particle (SRP) seems to be involved in a co-translational protein targeting mechanism conserved from bacteria to mammals [16,17,18]. In mammals, SRP interacts with the ribosome nascent chain complex (RNC) to translocate proteins across the endoplasmic reticulum membrane. The entire complex links with an SRP-receptor complex to be attached to the membrane [19].

The *E. coli* SRP mechanism comprises a 4.5S RNA (scRNA), which is the fifty-four homolog protein (Ffh) that is homologous to the mammalian SRP54 protein. It is also homologous to the FtsY protein, which has a C-terminal domain 300-residues long that is similar to the alpha subunit of the mammalian SRP receptor [20]. The interaction with the nascent pre-protein chain takes place via the M domain located at the Ffh carboxyl end. The minimal functional SRP conserved consists of Ffh and the scRNA [21]. The signal for targeting FtsY to the membrane is contained in the A domain located at the FtsY amino end [22,23,24]. SRP is responsible for targeting *E. coli* integral membrane proteins [25,26] and the export of *E. coli* SecB independent secretory proteins takes place without the postulated intervention of SRP [27]. The *E. coli* SRP based and the SecB-based targeting systems are substrate specific [28] and all the *E. coli* SRP components are essential for cell growth.

The *B. subtilis* SRP contains the scRNA, Ffh, and the histone-like protein HbsU [29]. The role of HbsU is still unknown. Although some studies indicated that the SRP could target *B. subtillis* secretory proteins via the Sec pathway [30], other authors have suggested that SecA could have a dual function in which it targets the newly made secretory protein to the membrane and pushes it through the translocase [30,31]. Therefore, there is a lack of fully convincing evidence showing whether, in the absence of SecB, the *B. subtilis* SRP could target both membrane and secretory proteins. The *S. lividans* SRP system consists of Ffh and an 82 nt long small size RNA (scRNA) [32]. The receptor protein FtsY forms part of the system and the three components are apparently expressed throughout cellular growth. No viable mutants in any of these three components have been obtained, and a possible essential role has been assigned to each of them. Experimental evidence obtained by co-immunoprecipitation studies has shown that *S. lividans* SRP is involved in targeting model secretory proteins [32,33]. The level of the SRP components seems to not be a limiting factor for the overproduction of secretory proteins in *S. lividans*, according to the results obtained with overproduced model proteins [34]. The *S. lividans* FtsY hydrophobic N-terminal segment identifies the protein as a homolog to other integral FtsY-like membrane proteins from other Actinobacteria with similar hydrophobic profiles [35]. The *S. lividans* strain is defective in the major type I signal peptidase (SipY; [34]) and has provided a useful tool for temporarily blocking translocation favoring the accumulation of pre-protein linked to SRP at the membrane. Therefore, it is helping detect the SRP ability to escort the pre-protein to the translocase complex [33]. This SipY deficiency also favored detecting an in vivo interaction of the SRP with a soluble form of FtsY, which acts as a functional cytoplasmic SRP receptor [36].

**Figure 1:**
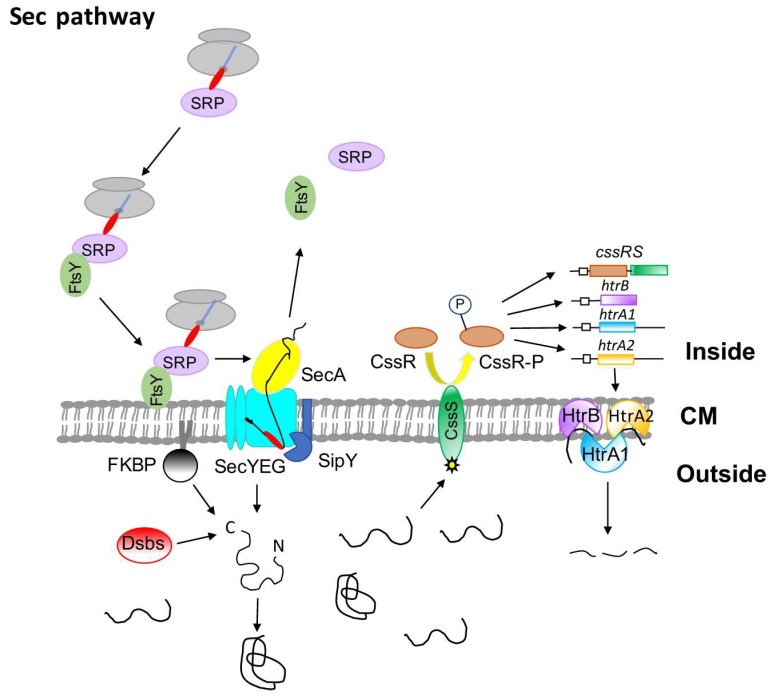
*Streptomyces lividans* major secretory route (Sec pathway). The secretory protein precursor is targeted to the membrane translocation complex by SRP, which is a GTP-dependent process, that requires the help of the FtsY receptor. When the signal peptidases (exemplified by the major signal peptidase, SipY) act, the SRP components are released to be used again. The extracellular presence of incorrectly folded mature proteins triggers the expression of the CssRS two-component system, which, in turn, induces the synthesis of the three HtrA-like proteins that are working cooperatively and degrades the incorrectly folded extracellular proteins. The acquisition of an active, correctly folded structure may need the action of enzymes involved in the formation of disulfide bonds (Dsbs) and/or peptidyl-prolyl cis-trans isomerases (FKBP-like).

In *E. coli*, SecY, SecE, and SecG form a heterotrimeric stable complex that gives rise to the channel that conducts the protein to be translocated [8] and the translocation of the *Streptomyces* secretory proteins is thought to take place in *E. coli*. Streptomycetes appear to have only one gene for each of these Sec proteins in their genomes including SecA [15]. In vitro experiments showed that *S. lividans* SecA was required for secretion of the Sec-dependent alpha-amylase [37], which suggests a comparable functioning of the *E. coli* equivalents. *S. coelicolor, S. lividans* [38], and *B. subtilis* [39] encoded SecG proteins have a functional homology to that of *E. coli*. Deletion of the *E. coli* or *B. subtilis* SecG causes a cold-sensitive phenotype while this seems not to occur in *S. lividans* [39,40]. Protein secretion is impaired in the *S. lividans* SecG deficiency, which delays the extracellular appearance of model secretory enzymes [38,41]. In *E. coli*, the heterotrimeric membrane complex formed by SecD, SecF, and YajC associates with the SecYEG channel, although YajC does not seem to be required for the complex to function [8]. The *S. coelicolor* genome contains two sets of SecD and SecF homologs required for an efficient secretion of some proteins [42]. Genes homologous to *yajC* have been identified in eleven *Streptomyces* genomes [43] even though a homolog to this does not seem to be present in the *S. lividans* 66 genome [3]. The functionality of these genes has not been determined experimentally. The YidC protein is essential in *E. coli* since it helps insert membrane proteins in a Sec-dependent [44] or a Sec-independent manner [45,46]. Two genes encoding potentially equivalent YidC proteins seem to form part of the *S. lividans* genome [3], where the YidC protein acts in *Streptomyces* in a Sec-dependent manner [47]. Figure 1 shows a scheme of how the Sec pathway works.

## 3. The Twin-Arginine (Tat) Pathway

The Tat route is an export pathway found in the thylakoid membranes of plant chloroplasts and described in several bacteria [48]. Proteins using this route appear to be secreted across the cytoplasmic membrane in a folded state [48]. This feature confers to the Tat pathway a particular interest to be exploited for the overproduction of secretory proteins in streptomycetes. The Tat pathway seems to consist of four proteins in *E. coli* including TatA, TatB, TatC, and TatE. The TatB-TatC complex binds to the signal peptide in an energy-independent manner to target the pre-protein to the membrane where Tat A polymerizes to form the channel for the extracellular protein to exit the cell. TatE has been shown in vivo and in vitro to interact with the translocase complex and is considered to be a regular element of the *E. coli* translocase [49]. In *B. subtilis* the TatB component seems to be absent and the Tat machinery is formed by three TatA subunits (TatAc, TatAd and TatAy) and two TatC subunits (TatCd and TatCy). In addition, two independent acting complexes with different substrate specifity are formed (TatAd-TatCd and TatAy-TatCy) and remain ambiguous to the TatAc function [50,51,52,53].

**Figure 2:**
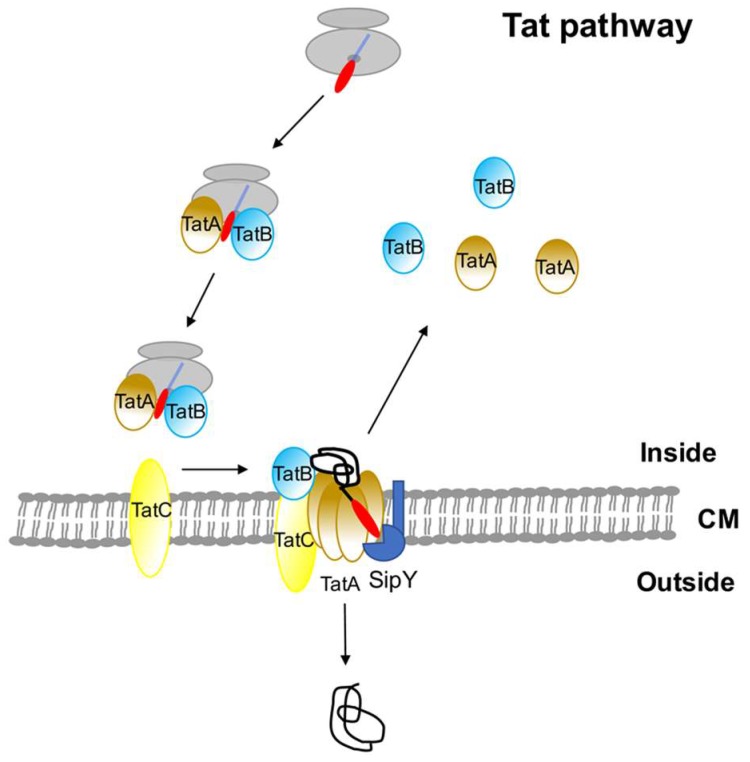
*Streptomyces lividans* minor secretory route (Tat pathway). The nascent secretory pre-protein using the Tat route is thought to be targeted to the membrane by the heterodimeric TatA-TatB complex. The signal peptide is cleaved by the signal peptidases (exemplified by the major signal peptidase, SipY) before releasing the mature protein to the cell wall.

*Streptomyces lividans* contains three Tat components (TatA, TatB, and TatC) with two different *tatA* genes [54]. Recognition of the Tat pre-secretory proteins in *S. lividans* is carried out by the heterodimeric complex formed by TatA and TatB, which theoretically target the pre-proteins to the membrane [55]. This heterodimer complex interacts with TatC seated in the membrane for the pre-proteins insertion. Then TatA oligomerises form the pore used by the folded secretory protein to leave the cell [55]. The eubacteria signal peptide of Tat peptides has some characteristic features that differentiate them from those of the Sec proteins. The n-region of the Tat signal peptide contains a conserved motif R-R-x-U-U where R-R is the twin-arginine motif and x and U are usually polar and hydrophobic amino acids, respectively [56,57,58]. Their h region is less hydrophobic and the c-region may contain basic residues [59]. The algorithms designed to predict the existence of this type of signal peptides identified a large number of potential Tat proteins (145–189) in *S. coelicolor* [60,61]. When membrane proteins from TatC deficient strains were compared to those of the isogenic wild type strain, only 27 proteins were unequivocally defined as Tat-dependent [58]. The algorithms predicted up to 127 Tat proteins in *S. lividans* [62]. However, the final number has not yet been experimentally determined even though the DypA peroxidase has been found to be a *S. lividans* Tat-dependent protein in this bacterium [63]. The annotated sequence of the *S. coelicolor* genome predicts more than 800 secretory proteins [15]. Therefore, in comparison, the assigned 27 Tat proteins involve a small number that reflects the minor role played by the Tat route in secretion. However, these 27 Tat proteins still represent a significant number when compared to those found in other bacteria. This reinforces the interest in exploring the Tat route for the secretion of correctly folded proteins in streptomycetes. The short number of predicted *E. coli* Tat proteins seems to bind redox cofactors, which are important for respiratory metabolism [64] while only three of the 27 experimentally confirmed *S. coelicolor* Tat-dependent proteins may bind cofactors. This reveals that the streptomycetes Tat pathway can export a broader spectrum of proteins than other bacteria [58]. Figure 2 illustrates the *S. lividans* Tat secretion pathway.

## 4. Stresses Caused by Overproduction of Secretory Proteins in Streptomycetes

The engineering of streptomycetes strains to overproduce homologous or heterologous secretory proteins require more in-depth knowledge of the potential detrimental effects that this overproduction may cause to the cell. Therefore, acquiring the best possible understanding of the physiological limiting steps that could be encountered when engineering bacterial secretory protein factories is optimal.

### 4.1. Temporal Translocase Blockage

Mutations in the *S. lividans* translocase component SecG or in the major signal peptidase SipY lead to a temporal translocation blockage in which the extracellular presence of several secretory factors are considerably reduced in both mutants, which is determined by the corresponding proteomics analyses and is consistent with the transcriptional profiles in each case [65]. The accumulation of unprocessed pre-proteins in the translocase complex generates a membrane stress in the SipY and SecG deficient mutants. A large number of genes encoding secretory proteins were down-regulated in the SipY deficient strain than in the SecG one, which was determined by transcriptional analyses and is in agreement with the proteomics results. This suggests a more dramatic effect of the SipY mutation than that of the SecG one. The formation of the aerial mycelium involves the action of extracellular proteins [12,66]. Temporal blockage of the pre-protein processing method would likely result in a severe limitation in efficiency protein secretion, which may lead to malfunction of the aerial mycelium formation and a deficient sporulation (the so-called bald phenotype). Comparison of the wild type and the mutant strains transcriptomic profiles identified a different set of bald-related genes down-regulated in each mutant strain in which a morphological analysis of the mycelial filaments under the scanning electron microscope showed that the effect was more dramatic in the SipY that in the SecG one. The differences in the expression of these bald-related genes together with the differences detected on the mutant strains extracellular proteins when compared to those of the wild type revealed by tandem mass spectrometry analysis constitute a characteristic profile of the “translocation stress” caused in the bacterial cell when the natural translocation process is impaired [65].

### 4.2. Induction of the Stringent Response

Bacteria frequently use a variety of sensor kinase genes contained in their genomes to respond to changes in the environment. The two-component environmental sensor systems are well represented in the streptomycetes genomes. A two-gene operon has been identified among the *S. coelicolor* sixty-seven two-component systems [67] that shares homology with the *B. subtilis degS-degU* operon, which regulates synthesis of some secretory proteins and the level of antibiotic production. This temporarily modulates the secondary metabolism of the bacterium [68,69]. Transcriptomic and tandem mass spectrometry analyses of the two-component operon defective strain as well as the strain harboring the regulator gene in high copy number allowed us to conclude that, when present in a high copy number, the regulator gene enhances antibiotic production and sporulation in early times of growth and considerably increases the expression of genes encoding the secretory protein to such an extent that the cellular amino acid pool suffers from depletion. This precursors deficiency triggers a stringent response in the bacterium, which mainly down regulates ribosomal genes [67]. Propagation of the regulator gene in high copy number could be useful if associated with overproduction of homologous or heterologous extracellular proteins in *Streptomyces*, but, at the same time, clearly points out the danger of provoking amino acid depletion, which may affect cell viability.

Lipoproteins are membrane-translocated proteins that remain associated with the cytoplasmic membrane. Bacterial lipoproteins are known to be involved in a number of essential cellular processes since most of the solute binding proteins are lipoproteins. They also take part in the cell envelope biogenesis process and in signal transduction pathways. In Gram-positive bacteria, a fair number of peptidyl-prolyl isomerases are lipoproteins responsible for the correct folding of extracellular proteins [70]. The pre-lipoproteins have a type II signal peptide that is specifically cleaved by a type II signal peptidase (Lsp) for which the presence of an essential sequence motif is necessary for the correct processing of the protein. After the translocation has taken place, a diacylgycerol transferase lipoprotein (Lgt) links a lipid molecule at the conserved cysteine residue where Lsp will cleave the signal peptide. Proteins homologous to Lgt have been found in the *S. coelicolor* and *S. lividans* genomes [3,71]. Proteins homologous to the N-acyl transferase Lnt add more lipids to the conserved cysteine residue in Gram negative bacteria and have also been described in streptomycetes strains. However, their deletion seems not to cause any effect to the *S. scabies* bacterium [72]. Most of the bacterial lipoproteins are Sec-dependent but some use the Tat system instead [71].

**Figure 3:**
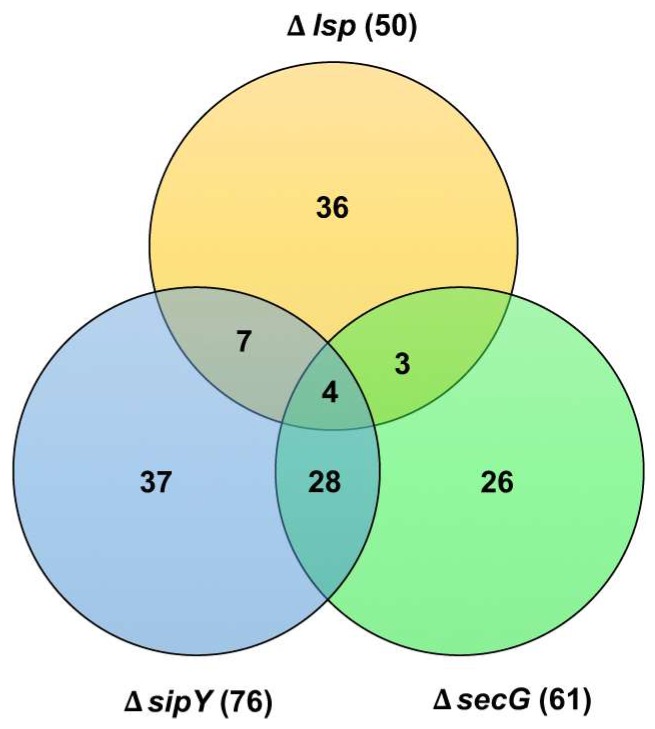
Venn diagram summarizing the relative degree of coincidence among the genes transcriptionally affected in the *Streptomyces lividans* SipY, SecG, or Lsp deficient strains. For comparative purposes, the set of genes involved in the stringent response in the Lsp deficient strains have been subtracted.

The transcriptomic study of Lsp-deficient *S. lividans* strain revealed that translocase blockage takes place, which the consequent negative effect on extracellular protein appearance causes sporulation delay and triggers the bacterial translocation stress response since the translocation is blocked by the accumulation of unprocessed lipoproteins [73]. This phenotype is observed when the cells were defective in the major type I signal peptidase, SipY, or the translocase SecG protein. Additionally, Lsp deficiency caused depletion of solute binding lipoproteins, which caused the depletion of nutrients inside the cell and triggered a stringent response [73]. This mimicked the effect caused by overproduction of secretory proteins when the regulator gene of the two-component operon described above was propagated in a high copy number [67]. These results are in accord with the key roles played by bacterial lipoproteins and they will have to take into account in the optimization of *Streptomyces* strains for the overproduction of secretory proteins. A Venn diagram [74] illustrates the relative degree of coincidence among the genes transcriptionally affected in the SipY, SecG, or Lsp deficient strains (see Figure 3). Therefore, 52.45% of the total genes regulated in the SecG deficient cells coincide with 42.10% of the total genes regulated in the SipY deficient cells. Lsp deficient cells show a coincidence on the 22.0% and the 14.0% of its genes with the 14.47% and the 11.47% of the genes regulated by the SipY and SecG deficiencies, respectively. Most of the affected genes were involved in nitrogen/amino acids metabolism, morphological differentiation and genes encoding a possible secreted protein that were downregulated in most cases (Table S1). Therefore, a selected subset of these genes could eventually be used to monitor an efficient secretory protein production in streptomycetes.

## 5. Overproduction of Model Secretory Proteins in *S. lividans*

The comparison of transcriptomic analyses of *S. lividans* strains overproducing secretory proteins via the Sec (alpha-amylase) or the Tat route (agarase) in *S. lividans* resulted in different cellular responses [75]. Basically, overproduction of the Tat model protein elicited the characteristic down regulation of genes involved in the stringent response while the same set of genes was upregulated when the Sec model protein was overproduced. The stringent response may cause cellular death and its potential induction has to be taken into account when engineering extracellular protein production in streptomycetes. Therefore, despite the potential attractiveness of using the Tat secretory route to obtain a potentially correctly folded extracellular protein, the Sec pathway could be the preferred route for secretory protein production in *Streptomyces* strains.

Additionally, some proteins appear to be able to use both routes. Therefore, when the Tat model protein agarase is overproduced in *S lividans*, a small amount of it was apparently secreted at the early phase of growth at a time when the Tat pathway is not functional, which was determined by co-immunoprecipitation studies using antibodies against agarase and the SRP components. This suggests that the protein could eventually be secreted via the Sec route [32]. A similar account has also been described for *E. coli* in which Tat signal peptides could trigger proteins to the Sec pathway [76] and the first two transmembrane domains of a Tat-membrane protein could be recognized by the Sec pathway. Sec and Tat routes seem to cooperate for the integration of a particular membrane protein [47]. Some membrane proteins with extracytoplasmic domains apparently need the cooperation of the Tat and Sec pathways [77].

The construction of chimeric precursor proteins interchanging the Sec-dependent alpha-amylase and the Tat-dependent agarase signal peptides showed that agarase was able to use the Sec route when led by the alpha-amylase signal peptide. However, the alpha-amylase did not succeed in using the Tat route led by the agarase signal peptide [41]. The inability of alpha-amylase to use the Tat signal peptide argues in favor of other parts of the protein apart from the signal peptide contributing to the streptomycetes secretion as described in *E. coli* [78]. The structure of the proteins may have evolved to select a particular secretory route. Most of the *S. coelicolor* unequivocally assessed Tat proteins [58] do not have predictable disulphide bonds while the major Sec proteins have at least one disulphide bond [65]. Structurally less complex secretory proteins may be engineered to use either secretory route while structurally complex proteins would have to be processed via the Sec pathway. Therefore, the Sec route may be the one to choose to overproduce extracellular proteins in streptomycetes.

## 6. Extracellular Stability of Secretory Proteins

Overproduction of secretory proteins in streptomycetes, as in other bacteria, could likely result in the extracellular accumulation of misfolded secreted proteins. In practical terms, they are inactive contaminants of the correctly folded, functionally active secreted proteins present in the total population of the overproduced ones. Therefore, previous identification and characterization of these quality factors are required in order to optimize extracellular protein production via the Sec route.

The *S*. *lividans* CssRS two-component system regulates the degradation of accumulated incorrectly-folded extracellular proteins outside the cell known as the secretion stress response. This two-component system is known to induce the synthesis of specific proteases to degrade the incorrectly-folded proteins and it has been described in *B. subtilis* [79] and *S. lividans* [80].

Overproduction of the *B. amyloliquefaciens* alpha-amylase in *B. subtilis* results in phosphorylation of the CssR regulator, which activates the expression of the genes *htrA* and *htrB* encoding two HtrA-like proteases [81,82]. An equivalent CssRS two-component system has been described in *S. lividans* [81]. When activated by the presence of incorrectly-folded alpha-amylase outside the bacterial cell, the regulator induced the expression of three different HtrA-like proteases encoding genes, *htrA1, htrA2*, and *htrB* ([83]; Figure 1). The CssR operon is a main element of the bacterial secretion stress response [80,84].

Genes encoding HtrA-like proteins are present in bacterial genomes and a significant number of bacteria have more than one HtrA-like protease [85]. HtrA proteins normally assemble into complex oligomers mainly via their PDZ domains in the carboxyl terminal part of the protein involved in substrate recognition, regulation of protease activity, and protein-protein interaction [86].

The *S. lividans* HtrA-like proteases seem to act in a cooperative manner since the alpha-amylase activity detected in each of the individual deficient strains was severely reduced when compared to the wild type one. A functional model for the combined action of the *S. lividans*’ three proteases has been proposed ([83]; Figure 1).

Different ways of action have been described for HtrA-like proteins in different bacteria and it is clear that, whichever they work, the final outcome is the degradation of the incorrectly-folded proteins present outside the cell that result from Sec-dependent secretion. Theoretically, secretory proteins using the Tat pathway should not need to activate the synthesis of HtrA-like proteins since these are secreted in an active conformation (see Figure 2).

Degradation of the extracellular accumulated incorrectly-folded secretory proteins becomes necessary to avoid their possible interference with essential bacterial cell processes and it is an effective way to correct that potentially harmful situation. An additional efficient way to deal with this accumulation is to procure other means to intervene in the correct folding of Sec-dependent proteins such as the action of the peptidyl-prolyl-isomerases (FKBP in Figure 1) and/or or thiol-disulfide oxidoreductases (Dsbs in Figure 1) equivalent to those described in many bacteria. A lipoprotein with peptidyl-prolyl isomerase activity likely involved in the folding of secretory proteins has been identified in *S. coelicolor* [71]. The mode of action of all these streptomycetes folding proteins still remain to be fully elucidated.

Exploring the *Streptomyces* response to different stresses that could potentially take place when a protein is overproduced has helped identify potential drawbacks in the secretory protein production as those described above, which facilitates a means to avoid them. Despite the potential existence of these stress responses, the actual amount of accumulated knowledge on how the streptomycetes protein secretion takes place and the potential use of the Table S1 listed genes to monitor the scale-up of secretory production points to *S. lividans* as a sufficient candidate for engineering the overproduction of extracellular proteins at the industrial level.

## Supplementary Materials

The following are available online at http://www.mdpi.com/2079-6382/7/2/33/s1, Table S1: Genes commonly modulated by the translocase blockage.
