# Supplementary material for: The Cellular Mechanisms that Ensure an Efficient Secretion in Streptomyces
Source: Antibiotics (Basel). 2018 Apr 14;7(2):33. doi: 10.3390/antibiotics7020033 (PMC6022935; doi:10.3390/antibiotics7020033)
Supplement: Supplementary file 1 [file antibiotics-07-00033-s001.pdf]

Table S1. Genes commonly modulated by the translocase blockage

Gene*	Annotated function	Regulation	Strain
Nitrogen/amino acis metabolism			
SLI0218	*narJ2*, nitrate reductase delta chain	Down	*sipY* and s*ecG* mutant
SLI0219	*narI2*, nitrate reductase gamma chain	Down	*sipY* and s*ecG* mutant
SLI1454	Possible amino oxidase	Up	*sipY* and s*ecG* mutant
SLI1498	*aroE*, shikimate 5-dehydrogenase	Down	*sipY* and s*ecG* mutant
SLI3073	*hutU*, urocanate hydratase	Up	*secG* and *lsp* mutant
Oxidative stress/osmotic stress			
SLI1866	Putative condensing enzyme	Up	*sipY* and *lsp* mutant
SLI5254	*sodN*, superoxide dismutase	Down	*sipY* and *lsp* mutant
Membrane/transport protein			
SLI5123	Putative small membrane protein	Down	*sipY* and *lsp* mutant
Morphological differentiation			
SLI0381	Possible glycosyl transferase	Down	*sipY* and s*ecG* mutant
SLI0382	Probable UDP-glucose/GDP-mannose	Down	*sipY* and s*ecG* mutant
	family dehydrogenase (putative secreted		*sipY* and s*ecG* mutant
	protein)		
SLI0383	Hypothetical protein (possible target for	Down	*sipY* and s*ecG* mutant
	bldA regulation)		
SLI0392	Possible methyltransferase similar to *S.*	Down	*sipY* and s*ecG* mutant
	peucetis daunorubicin/ doxorubicin		
	enzyme DnrX	Down	
SLI0393	Possible transferase	Down	*sipY* and s*ecG* mutant
SLI0712	*lipR* putative transcripcional activator	Down	*sipY* and s*ecG* mutant
SLI1630	*cvnA9*, *rarA* putative integral membrane	Down	*sipY* and s*ecG* mutant
	protein		
SLI1674	*chpC*, possible small membrane protein	Down	*sipY* and s*ecG* mutant
SLI1675	*chpH*, possible small membrane protein	Down	*sipY* and s*ecG* mutant
SLI1800	*chpE*, possible small membrane protein	Down	*sipY* and s*ecG* mutant
SLI2699	*chpG*, putative small membrane protein	Down	*sipY* and s*ecG* mutant
SLI2705	*chpF*, possible membrane protein	Down	*sipY* and s*ecG* mutant
SLI2717	*chpD*, putative small membrane protein	Down	*sipY* and s*ecG* mutant
SLI3323	*bldN*, probable RNA polymerase sigma	Down	*sipY* and s*ecG* mutant
	factor		
SLI3549	*bldG*, probable anti-sigma factor	Down	*sipY* and s*ecG* mutant
	antagonist		
SLI4768	*bldM*, probable two-component regulator	Down	*sipY* and s*ecG* mutant
SLI5113	*bldKB*, putative ABC transport system	Up	*sipY* and s*ecG* mutant
	lipoprotein		
SLI5114	*bldKC*, putative ABC transport system	Up	*sipY* and s*ecG* mutant
	integral membrane protein		*sipY* and s*ecG* mutant
Glicerol metabolism and glycolysis			
SLI1659	*glpF*, probable glycerol uptake facilitator	Up	*sipY* and s*ecG* mutant
	protein		
SLI1660	*glpK*, glycerol kinase	Up	*sipY* and s*ecG* mutant
Secondary metabolites			
SLI6073	*geoA*, possible cyclase germacradienol/	Down	*sipY* and s*ecG* mutant
	geosmin synthase		
Secreted proteins			
SLI0297	Possible secreted protein	Down	*sipY*, *secG*, *lsp* mutant
SLI0736	Possible secreted protein	Down	*secG* and *lsp* mutant
SLI0762	*sti1*, protease inhibitor	Down	*sipY*, *secG*, *lsp* mutant
SLI1860	Possible secreted protein	Down	*sipY*, *secG*, *lsp* mutant

SLI2116	Possible secreted protein	Down	*secG* and *lsp* mutant
SLI6197	Possible secreted protein	Down	*sipY* and *lsp* mutant
SLI6198	Possible secreted protein	Down	*sipY* and *lsp* mutant
SLI7657	Possible secreted protein	Down	*sipY, secG, lsp* mutant
Other genes			
SLI0682	Hypothetical protein	Down	*sipY* and s*ecG* mutant
SLI2822	cvnD12, posible conserverd ATP/GTP-	Down	*sipY* and *lsp* mutant
	binding protein		
SLI6393	Probable transposase IS21/IS1162 family	Down	*sipY* and s*ecG* mutant
SLI7658	Hypothetical protein	Down	*sipY* and *lsp* mutant

*Genes names are as appeared in references [64,73].
